# Multiple copies of BCR-ABL fusion gene on two isodicentric Philadelphia chromosomes in an imatinib mesylate-resistant chronic myeloid leukemia patient

**DOI:** 10.3892/ol.2013.1225

**Published:** 2013-03-05

**Authors:** WALID AL-ACHKAR, ABDULSAMAD WAFA, FATEN MOASSASS, ELISABETH KLEIN, THOMAS LIEHR

**Affiliations:** 1 Department of Molecular Biology and Biotechnology, Human Genetics Division, Atomic Energy Commission, Damascus, Syria;; 2 Jena University Hospital, Friedrich Schiller University, Institute of Human Genetics, D-07743 Jena, Germany

**Keywords:** chronic myeloid leukemia, isodicentric Philadelphia chromosomes, fluorescence *in situ* hybridization, imatinib mesylate

## Abstract

The so-called Philadelphia (Ph) chromosome is present in more than 90% of chronic myeloid leukemia (CML) cases. Amplification or duplication of the BCR-ABL gene has been found to be one of the key factors leading to drug resistance to imatinib mesylate (IM). In the present study, we identified the presence of isodicentric Ph chromosomes [idic(Ph)] in an IM-resistant patient. Fluorescence *in situ* hybridization (FISH) analysis on metaphase chromosomes confirmed the heterogeneity and amplification of the fused BCR-ABL gene. FISH analysis superimposed on G-banding confirmed the presence of idic(Ph) chromosomes. Reverse transcription-polymerase chain reaction (RT-PCR) products revealed the presence of the BCR-ABL fusion transcript b3a2. The idic(Ph) chromosomes in CML were shown to be fused at the satellite regions of the short arms. The patient did not respond to IM chemotherapy and did not achieve remission. In this study, the impact of the idic(Ph) chromosomes on genomic instability, heterogeneity and amplification of the BCR-ABL gene in IM-resistant patients is discussed.

## Introduction

Chronic myeloid leukemia (CML) is an acquired myeloproliferative disorder that originates in an abnormal pluripotent bone marrow stem cell and is consistently associated with the presence of the Philadelphia (Ph) chromosome, usually leading to a BCR-ABL gene fusion. The Ph chromosome is the result of a balanced t(9;22)(q34;q11) translocation, and is observed in more than 90% of CML cases, with variant Ph translocations being observed in the remainder (1). The BCR-ABL fusion gene is formed by the transposition of the 3’ portion of the ABL oncogene from 9q34 to the 5’ portion of the BCR gene on chromosome 22, and this fusion gene encodes a constitutively active tyrosine kinase (2).

The progression of CML from the chronic phase (CP) to blast crisis (BC) is frequently associated with non-random secondary chromosomal aberrations, including +8, i(17q), +19 and an extra Ph chromosome (3).

The isodicentric Ph chromosome [idic(Ph)] is a rare cytogenetic aberration formed by the duplication and fusion of two identical Ph chromosomes with retention of their centromeres. Idic(Ph) chromosomes have been previously observed in CML patients (4–10).

Targeted therapy has been realized with imatinib mesylate (IM) (Glivec, formerly STI571), which forms a complex with the ABL part of the fused gene and inactivates it (11). IM is a highly effective therapy that has demonstrated a complete cytogenetic response in 87% of patients with newly-diagnosed CP CML (12). A complete hematological response with IM therapy has been observed in 95% of patients with CP CML following failure of interferon-α, 71% of accelerated phase (AP) patients and 31% of patients in myeloid blast crisis (BC) (13–15).

Resistance to chemotherapy occurs as a result of increased expression of the BCR-ABL kinase from genomic amplification, clonal chromosomal evolution, or mutations in the ABL kinase of the BCR-ABL gene affecting drug interaction or kinase activity (16).

In the present study, we describe a rare case of isoderivative Ph chromosome [ider(22)]-positive CML, which was further characterized by fluorescence *in situ* hybridization (FISH) and reverse transcription-polymerase chain reaction (RT-PCR). The patient did not respond to IM chemotherapy.

## Materials and methods

### Case report

A 33-year-old male was diagnosed as suffering from CP CML. In May 2010, the white blood cell (WBC) count of the patient was 25.5×10^9^/l, consisting of 78.5% neutrophils, 16.8% lymphocytes and 4.7% monocytes. The platelet count was 432×10^9^/l and the hemoglobin level was 14.1 g/dl. A previous physical examination revealed splenomegaly, loss of weight and fever. The patient was treated with IM at 400 mg/day for a total of 54 months, following which the previous relevant symptoms appeared to have improved. In July 2011, the patient presented for the second time with a WBC of 14.6×10^9^/l consisting of 46.1% neutrophils, 27.7% lymphocytes, 22.2% monocytes, 0.9% eosinophiles and 3.1% basophiles. The platelet count was 117×10^9^/l and the hemoglobin level was 13.3 g/dl. The serum lactate dehydrogenase (LDH) level was 613 U/l (normal level up to 414 U/l) and the serum alkaline phosphatase level was 83 U/l (normal level up to 128 U/l). The patient was treated with IM at 800 mg/day for a total of 6 months. The patient had a brother diagnosed with CML in 1994 who succumbed following 6 months of chemotherapy.

### Cytogenetic analysis

Chromosome analysis using GTG-banding was performed according to standard procedure (17). A total of 20 metaphase cells derived from the unstimulated bone marrow of the patient were analyzed. Karyotypes were described according to the international system for human cytogenetic nomenclature (18).

### Molecular cytogenetics

FISH using a LSI BCR-ABL dual-color dual-fusion translocation probe (Abbott Molecular/Vysis, Des Plaines, IL, USA) was performed according to the manufacturer’s instructions (17). Furthermore, a probe specific to all acrocentric short chromosome arms (midi54) was applied as previously reported (19). A total of 20 metaphase spreads were analyzed using a fluorescence microscope (AxioImager. Z1 mot, Zeiss, Hertfordshire, UK) equipped with appropriate filter sets to discriminate between a maximum of five fluorochromes and the counterstain DAPI. Image capturing and processing were carried out using an ISIS imaging system (MetaSystems, Altlussheim, Germany).

### Immunohistochemistry

The immunohistochemical tests were performed on Carnoy fixed cell suspension as previously described (20). A rabbit polyclonal to CENP-B antibody (Abcam, Cambridge, UK) was used to stain all centromeres. The specific staining of the active centromeres was performed with the anti-CENP-C antibody. FITC-labeled goat anti-rabbit IgG and CyTM3-conjugated AffiniPure goat anti-guinea pig IgG (Dianova, Hamburg, Germany) were applied as secondary antibodies.

### Reverse transcription-polymerase chain reaction (RT-PCR) for BCR-ABL fusion transcripts

RT-PCR was carried out as previously described (21).

## Results

**Figure 1:**
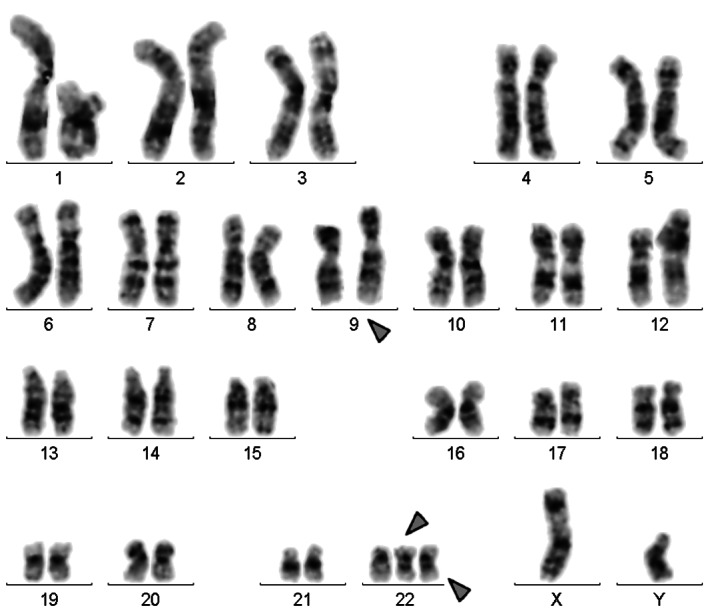
GTG-banding revealed a karyotype 47,XY,t(9;22),-22,+der(22) ×2[13]/46,XY,t(9;22)[7] involving one further chromosome in addition to chromosomes 9 and 22. Derivative chromosomes are indicated by the arrowheads.

**Figure 2:**
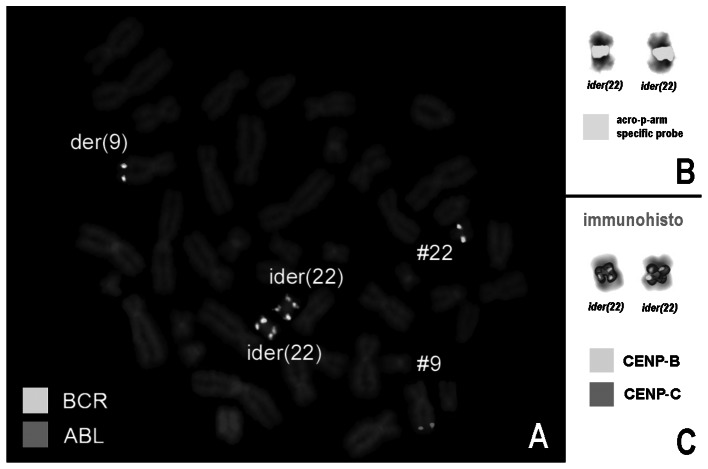
(A) Fluorescence *in situ* hybridization (FISH) using probes for BCR (green) and ABL (red) revealed 5 copies of BCR-ABL on the ider(22) chromosome in this case. (B) Using a probe for all acrocentric short arms, the breakpoints of ider(22) were shown to be located in the short arms. (C) Using immunohistochemistry, the dicentric character of the ider(22)s was proven. The CENP-B antibody stains all centromeres apart from that of the Y-chromosome, while the CENP-C antibody stains only active centromeres. Thus, it was also proven that the two centromeres were active on the ider(22). #, chromosome; der, derivative chromosome; ider, isoderivative chromosome.

Karyotyping was performed following the chemotherapy treatment, revealing the following karyotypic changes. A complex karyotype 47,XY,t(9;22),-22,+der(22)×2[13]/46,XY, t(9;22)[7] was determined by GTG-banding (Fig. 1) and was further specified by molecular cytogenetic studies (Fig. 2A and B). Dual-color FISH using a probe specific to BCR and ABL revealed that two atypical Ph chromosomes with four BCR-ABL fusion genes were present, i.e., there were two isoderivative chromosome 22 [ider(22)] and the final karyotype obtained was: 47,XY,der(9)t(9;22)(q34.13;q11.23),-22 +der(22)(9qter->9q34.13::22q11.23->22p12∼13::22p12∼13-> 22q11.23::9q34.13->9qter) ×2[13]/46,XY,t(9;22)(q34;q11)[7].

RT-PCR analysis of the fusion transcript revealed a band corresponding to the b3a2 transcript (data not shown).

Immunohistochemistry revealed that the two centromeres on ider(22) were active (Fig. 2C).

## Discussion

According to the literature, reports of idic(Ph) chromosomes in CML have been infrequent, with most of them formed as a result of a fusion in the short arm of chromosome 22. From a cytogenetic viewpoint, idic(Ph) chromosomes formed by fusion at band q11 have rarely been observed, and have been reported in CML (4–9) and in acute lymphoblastic leukemia (ALL) (10).

Idic(Ph) chromosomes appear to be created by fusion or translocation of double Ph chromosomes (6). Furthermore, in certain cases of CML, idic(Ph) chromosomes are also duplicated, and double or triple idic(Ph) chromosomes are found (4,6,7). The formation of idic(Ph) chromosomes may play a crucial role in the amplification and heterogeneity of the BCR-ABL gene, leading to resistance to IM therapy in CML patients (8). However, the resistance can be primary or secondary (following an initial response). The two types of resistance occur most frequently in the BC phase of CML (7). Multiple mechanisms of resistance to IM have been described (10).

Cytogenetic studies often provide evidence of the progression of disease at an earlier phase than hematological markers. It is known that the expression of BCR-ABL is elevated in progenitor cells in the BC phase, compared to the CP phase of CML (22). While the findings involving the peripheral blood and bone marrow of the patient were suggestive of CP CML, given the presence of multiple copies of the idic(Ph) chromosome, it was possible that cytogenetics indicated the progression of the disease towards an accelerated phase. Despite the absence of mutations in the drug-binding site, the presence of multiple copies of the BCR-ABL oncogene is indicative of a poor prognosis and higher possibility of resistance to drug treatment (23).

The causative factor in the formation of isodicentric chromosomes is unknown. Isodicentric chromosomes may lead to breakage and reunion cycles during mitosis, potentially forming ring chromosomes and thus leading to genomic instability and heterogeneity in the cell population. Furthermore, these isodicentric chromosomes may be heterogeneous in copy number, leading to the amplification and duplication of the hybrid BCR-ABL genes on the idic(Ph) chromosome. Gene amplification and genomic heterogeneity are known to be associated with drug resistance (6,24). To the best of our knowledge, no acquired dicentric chromosome has yet been studied for its centromeric activity. Notably, in the two ider(22) both centromeres were active. According to previous studies on inborn dicentrics the two centromeres are active if a distance between approximately 1.4 and 13 Mb is present; in case of distances larger than 15 Mb one centromere becomes inactive (20).

The problem of gene amplification and genomic instability may be overcome by administering higher doses of IM to patients who develop this subcategory of IM resistance (8). However, if IM is evident in the etiology of the chromosomal breakpoints, inducing non-random breakpoints at the subtelomere or telomere region of the Philadelphia chromosomes, alternative therapies should be investigated. The elucidation of these specific disease mechanisms may assist in yielding additional therapies, possibly to be co-administered with IM (8).

In conclusion, we reported a rare case of isoderivative Ph chromosome-positive CML in AP, which was further characterized by FISH and RT-PCR. The patient did not respond to IM chemotherapy.
